# Merkel Cell Polyomavirus and DNA Damage Response

**DOI:** 10.3390/ijms27198881

**Published:** 2026-10-06

**Authors:** Sara Passerini, Sara Messina, Ugo Moens, Valeria Pietropaolo

**Affiliations:** 1 Department of Public Health and Infectious Diseases, Sapienza University of Rome, 00185 Rome, Italy; sara.messina@uniroma1.it; 2 Department of Medical Biology, Faculty of Health Sciences, University of Tromsø-The Arctic University of Norway, 9019 Tromsø, Norway; ugo.moens58@gmail.com

**Keywords:** DNA damage response, Merkel cell carcinoma, Merkel cell polyomavirus, large T antigen, small t antigen, therapy

## Abstract

Cellular DNA is constantly exposed to endogenous and exogenous genotoxic factors causing mutations. Therefore, safeguarding the integrity of the human genome is a *conditio sine qua non* for normal cell function. To maintain genomic integrity, complex multiprotein networks, known as DNA damage response (DDR) signaling pathways, sense DNA lesions and repair mistakes or induce cell death when extensive unrepairable damage has occurred. Two of the major DDR pathways are the ataxia-telangiectasia mutated (ATM) and ataxia-telangiectasia and Rad3-related (ATR) cascades. The ATM pathway primarily responds to double-strand breaks, whereas the ATR cascade is activated by single-stranded DNA and mediates the response to replication stress. One of the exogenous factors that can induce DNA aberrations and trigger the ATM and ATR pathways is viral infection. This review focuses on current knowledge regarding the interaction between Merkel cell polyomavirus, a major etiological risk factor of Merkel cell carcinoma, and the ATM and ATR pathways and the effect on viral replication and virus-induced oncogenesis. The review elaborates on the role of the viral oncoproteins large T antigen and small t antigen in DDR interaction. Possible therapies targeting DDR in Merkel cell carcinoma and gaps in understanding the molecular mechanisms of Merkel cell polyomavirus-triggered ATM and ATR pathway activation are discussed.

## 1. The DNA Damage Response Signaling Pathway

The genome of cells is continuously exposed to damage evoked by endogenous and exogenous genotoxic stressors such as DNA replication, reactive oxygen species, chemical mutagens, ionizing radiation, and UV light, as well as bacterial, viral and parasitic infections [1,2,3,4,5]. To secure the integrity of the DNA, complex cellular mechanisms detect and sense the extent of DNA damage and then activate a cascade of processes that will either repair the DNA or induce cell death [6,7]. This complex network safeguarding the genome is called the DNA damage response (DDR). To address the wide variety of DNA lesions, the DDR encompasses multiple repair mechanisms, including Base Excision Repair (BER) for small chemical alterations of DNA bases; Nucleotide Excision Repair (NER), which detects helix-distorting DNA lesions; and Mismatch Repair (MMR) for replication errors. Moreover, Single-Strand Break Repair (SSBR) is involved in processing single-strand breaks (SSBs), whereas double-strand breaks (DSBs) are addressed by distinct pathways, including homologous recombination (HR) and non-homologous end joining (NHEJ) [6,7].

**Figure 1:**
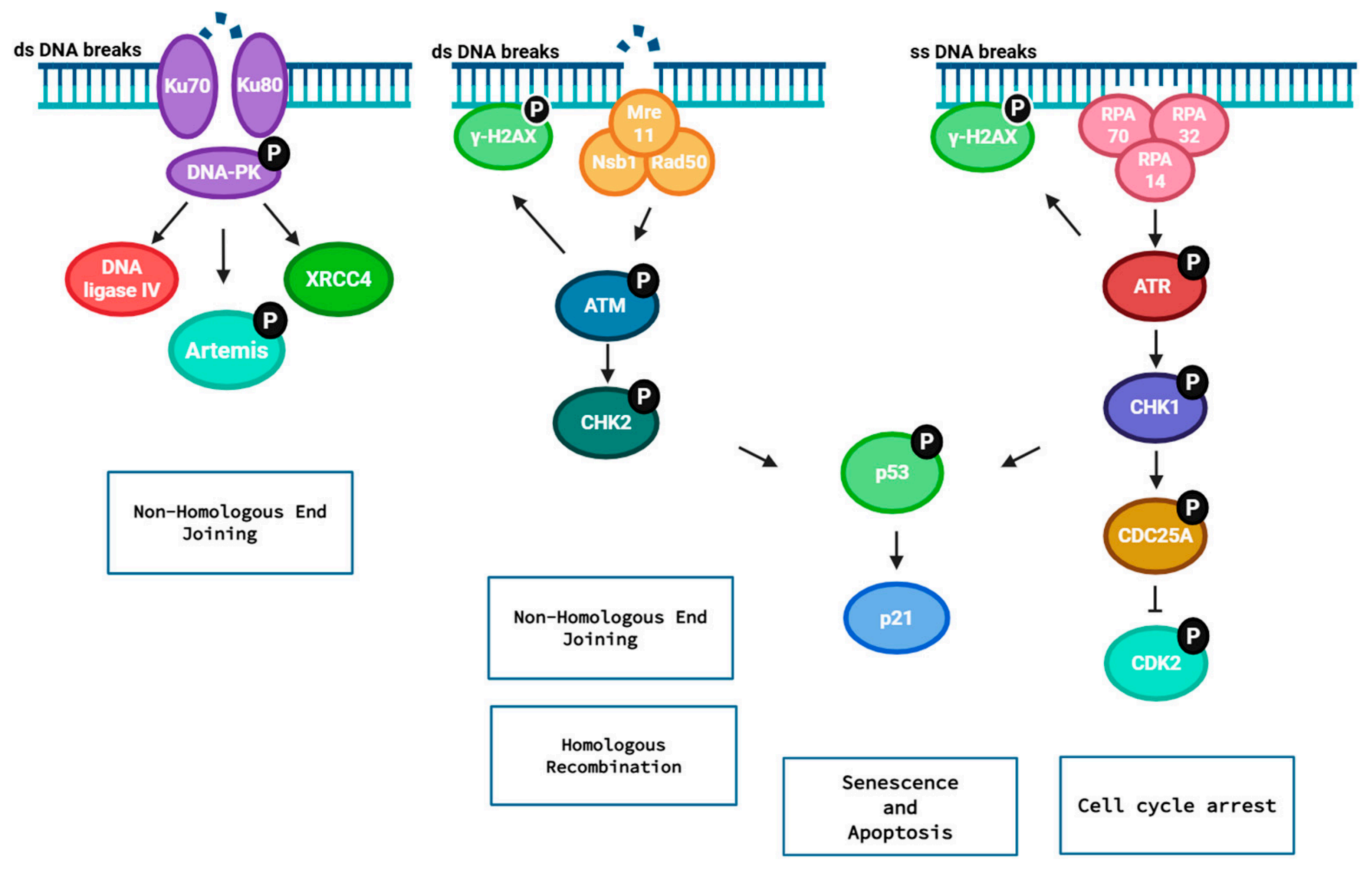
The major DNA damage response pathways. Exogenous and endogenous genotoxic stressors induce SSBs or DSBs. The ATM pathway is activated upon DSBs, which are detected by the MRN complex. This complex induces autophosphorylation of ATM, which in turn phosphorylates CHK2. Activated CHK2 then phosphorylates p53. DSBs also lead to activation of the DNA-PK pathway, which signals through CHK1 and CDC25A and CDK2 to induce cell cycle arrest. The ATR pathway is triggered by ssDNA, which is recognized by the RPA complex. ATR phosphorylates CHK1, which can then phosphorylate p53. ATM and ATR can both phosphorylate H2AX. See text for details. Created in BioRender (2026) https://BioRender.com/c45jc3v (accessed on 8 September 2026).

Key components in the DDR are the ataxia-telangiectasia mutated (ATM) pathway, the ataxia-telangiectasia and Rad3-related (ATR) pathway and the DNA-dependent kinase (DNA-PK) pathway [8,9,10] (see Figure 1).

The ATM pathway is activated upon DSB and mediates DNA repair [11,12]. DSBs are detected by the Mre11-Rad50-Nbs1 (MRN) complex, which induces autophosphorylation of ATM at serine (Ser) 1981 [13], and this activated Ser/Thr (threonine) kinase in turn phosphorylates its downstream target, the Ser/Thr protein kinase checkpoint kinase 2 (CHK2), at Thr-68 [14]. Activated CHK2 can then phosphorylate multiple target proteins, including p53 at, amongst others, Thr-18 or Ser-20 [15,16,17]. DSB repair is mediated by two main mechanisms: HR, which is closely regulated by ATM signaling, and NHEJ, which primarily relies on DNA-PK activity [11,12]. HR operates during the late S or G2 phases, when the sister chromatid is available as an intact template, and is therefore generally considered a high-fidelity repair pathway. In contrast, NHEJ occurs throughout the cell cycle and directly rejoins broken DNA ends. As it does not require a homologous template, the processing of DNA ends during NHEJ can result in small insertions or deletions, making it more error prone than HR [8]. 

Another major component of the DDR pathway is the ATR cascade, which plays a central role in the cellular response to DNA replication stress. ATR primarily responds to single-stranded (ss) DNA lesions [8,9,18,19]. Notably, single-stranded DNA (ssDNA) is recognized by the replication protein A (RPA) complex, which is built up of RPA70, RPA32 and RPA14. The RPA trimer activates the Ser/Thr kinase ATR by stimulating the intrinsic autophosphorylation activity of ATR. Activated ATR signals downstream through phosphorylation of CHK1 at Ser-317 and Ser-345 [15,17,20]. Activated CHK1 can phosphorylate p53 at multiple sites in the N- and C-terminal regions [15,17]. Phosphorylation of p53 by either CHK1 or CHK2 stimulates the transcriptional activity of p53. One of p53’s target genes is *CDKN1A*, which encodes the cyclin-dependent kinase inhibitor p21 [21,22]. p21 has an inhibitory effect on the cell cycle, can interfere with apoptosis, and is required for the efficient repair of different types of genotoxic lesions [23,24,25]. CHK1 can also phosphorylate cell division cycle 25A (CDC25A), a protein phosphatase, which removes inhibitory phosphates from cyclin-dependent kinase 2 (CDK2). Phosphorylation of CDC25A inactivates its phosphatase activity, and as a result, CDK2 remains inactive, leading to cell cycle arrest [26]. 

Another common substrate for ATM and ATR is the histone variant H2AX. In its Ser-139-phosphorylated form, referred to as γH2AX [27], this protein enables DNA unwinding, thereby facilitating DNA repair enzymes to correct the aberration in the DNA [28,29]. γH2AX is a well-known hallmark of DNA damage [30].

The DNA–protein kinase (DNA-PK) pathway repairs DSB through non-homologous end joining. DNA-PKcs is a holoenzyme with the catalytic subunit DNA-PK and the regulatory subunits Ku70 and Ku80 [9,10,31]. Activation of DNA-PK occurs through autophosphorylation. In addition, ATM and ATR can phosphorylate DNA-PK and contribute to its regulation. The holoenzyme DNA-PK binds to the broken DNA ends and recruits the endonuclease Artemis (which is phosphorylated by DNA-PK), X-ray cross-complementing protein 4 (XRCC4), DNA polymerase and DNA ligase IV to join the broken DNA ends [9,10,31]. 

In conclusion, activation of the ATM, ATR and DNA-PK pathways results in cell cycle arrest and DNA repair. If DNA damage is too extensive and unrepairable, the apoptosis pathway is turned on and the damaged cell is destroyed [7,8,9,19,32].

## 2. Human Polyomaviruses and Merkel Cell Polyomavirus

Polyomaviruses (PyVs) are small dsDNA viruses that are widespread in birds and mammals. Fifteen different polyomaviruses have been detected in humans so far: Simian Virus 40 (SV40 or Macaca mulatta polyomavirus 1), BKPyV, JCPyV, KIPyV (Karolinska Institutet polyomavirus), WUPyV (Washington University polyomavirus), MCPyV (Merkel cell polyomavirus), HPyV6, HPyV7, TSPyV (Trichodysplasia spinulosa-associated polyomavirus), HPyV9, HPyV10 (MWPyV or Malawi polyomavirus), HPyV11 (STLPyV or St. Louis polyomavirus), HPyV12, NJPyV (New Jersey polyomavirus), LIPyV (Lyon IARC polyomavirus) and QPyV (Quebec polyomavirus) [33,34,35]. However, not all are genuine human polyomaviruses. The authentic host for SV40 is the rhesus macaque [34], whereas shrews are the natural host for HPyV12 (hence, this virus was renamed Sorex araneus polyomavirus or Sara-PyV1) [36], and LIPyV seems to be a feline polyomavirus [37,38,39]. QPyV has not been assigned as a human polyomavirus by the International Committee on Taxonomy of Viruses [34]. 

Human polyomavirus (HPyV) infection occurs in early childhood, and several studies have shown that seroprevalence for all genuine HPyVs reaches ≥70% in the adult healthy population [40,41,42,43,44,45,46,47,48]. So far, seropositivity for QPyV has not been investigated. While primary infection seems asymptomatic and HPyVs appear to establish a lifelong harmless latent infection, some HPyVs can lead to severe diseases in certain circumstances, especially in patients who are immunocompromised (Table 1). BKPyV has been linked to PyV-associated nephropathy and hemorrhagic cystitis in patients who have undergone a renal transplant or bone marrow transplant, while JCPyV is linked to progressive multifocal leukoencephalopathy. TSPyV is associated with trichodysplasia spinulosa, and HPyV6 and HPyV7 may cause pruritic rashes and dyskeratotic dermatosis [49,50,51,52]. In vitro and in vivo studies with HPyV or viral proteins demonstrated that some have oncogenic potential [53], but only MCPyV has been firmly linked to human malignancy: the highly aggressive skin cancer Merkel cell carcinoma (see Section 3) [54,55,56,57]. BKPyV is associated with bladder cancer, and JCPyV forms an etiological risk factor for colorectal cancer and brain tumors [53,58,59,60]. The International Agency for Research on Cancer categorized MCPyV as a 2A agent (probably carcinogenic to humans), whilst BKPyV and JCPyV are considered 2B (possibly carcinogenic to humans) [61].

HPyVs have a circular dsDNA genome of approximately 5000 base pairs that is surrounded by a capsid, but an envelope is lacking [33]. Unlike the other HPyVs whose capsid consists of three different proteins, VP1, VP2, and VP3, the MCPyV capsid lacks VP3 [62].

**Figure 2:**
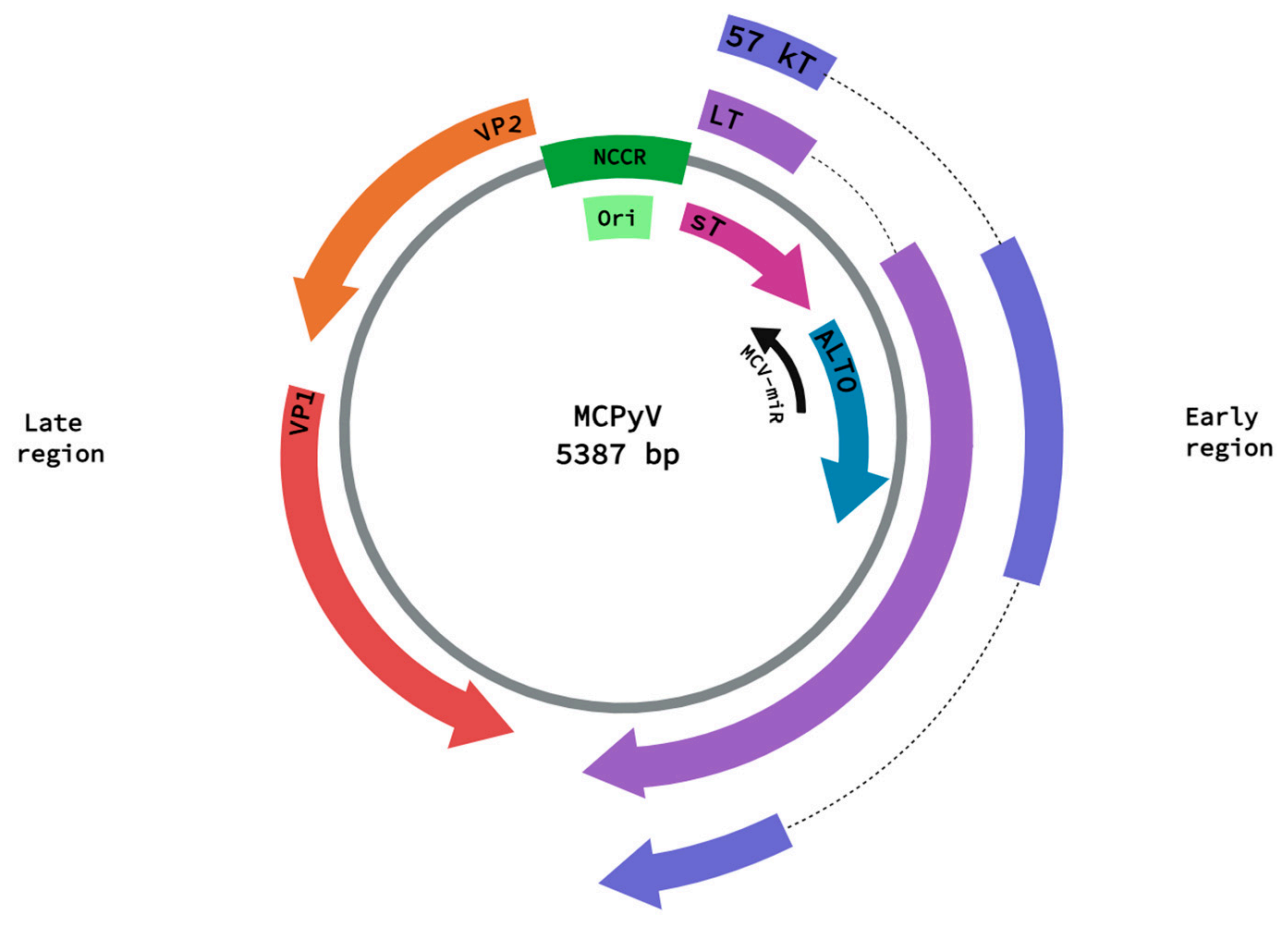
Representation of the MCPyV genome. See text for details. Created in BioRender (2026) https://BioRender.com/s3tvklk (accessed on 8 September 2026).

The HPyV genome consists of three functional domains. The early region encodes the regulatory proteins large T antigen (LTAg) and small t antigen (sTAg), which are involved in viral transcription and replication, and the late region encodes the capsid proteins. The early and late regions are separated by a non-coding control region (NCCR). This region contains the origin of replication and the transcription control elements for the early and late genes [63,64]. The early region of MCPyV encodes two additional proteins that have not been identified in other HPyVs (Table 1, Figure 2). One protein, referred to as ALTO (acronym for Alternate frame of Large T Open reading frame), does not seem to be required for viral replication because mutations abolishing the expression of ALTO did not affect viral replication [65]. The ALTO protein can also be generated through two virus-encoded circular RNAs: circALTO1 and circALTO2 [66]. ALTO was shown to upregulate the transcriptional activity of some cellular promoters [66]. Recent studies have shown that ALTO activates the NFκB and the STING-TBK1 (Stimulator of Interferon Genes - Tank-binding kinase 1) pathways. These interactions repress early gene transcription and viral replication and stimulate immune evasion, thereby promoting latency [67,68,69]. The function of the other protein, 57kT, remains elusive [70,71]. The MCPyV late region encodes the capsid proteins VP1 and VP2 and two microRNAs (mcv-miR-M1-5p and mcv-miR-M1-3p) that can suppress LTAg expression and target genes whose products are involved in immune evasion (Figure 2) [62,72,73]. 

## 3. Merkel Cell Polyomavirus and Merkel Cell Carcinoma

Merkel cell carcinoma (MCC) is an aggressive skin neuroendocrine tumor that typically occurs in elderly people. MCC malignancy is rare, with 0.7–2.5 new cases per 100,000 people annually depending on the geographic region. The 5-year survival rate is <45%, and the mortality rate varies between 14% (metastasis) and 51% (local disease), making it amongst the highest rates for skin cancer [57,74,75]. In 2008, the group of Chang and Moore identified a new polyomavirus in 80% of examined MCC cases and named it MCPyV [76]. Additional studies of MCC specimens from different parts of the world have confirmed MCPyV in 60–80% of cases, and the virus is now considered a major etiological factor of this malignancy [77]. Another cause of MCC is UV light, and MCC can therefore be subdivided into virus-positive or virus-negative cases [57,78,79]. Typical for UV-driven MCC is a significantly higher mutation burden than in virus-positive samples, whereas the latter are characterized by the integration of the MCPyV genome and expression of a C-terminal truncated LTAg [80,81,82,83,84].

Cell culture and animal studies with non-human polyomaviruses (e.g., mouse PyV and SV40) and HPyVs (e.g., BKPyV and JCPyV) have shown that LTAg is the major oncoprotein and that sTAg has an auxiliary function in cellular transformation [63,85,86]. For MCPyV, the situation seems to be the opposite, with sTAg as the major oncoprotein and LTAg exerting a supporting function [56,87,88,89,90]. The interaction of MCPyV LTAg and sTAg with several cellular proteins promotes cell mobility, migration, and metabolic perturbation and disturbs the cellular transcriptome and autophagy [71,91,92,93,94,95,96,97,98] and reviewed in the mentioned references [56,90].

Integration of the viral genome and expression of a C-terminal-truncated LTAg (tLTAg) caused by a nonsense mutation in the *LTAg* gene are hallmarks of MCPyV-positive MCCs [70,76]. The length of the tLTAg expressed in MCC tumors varies. Whereas full-length LTAg contains 817 aa, the shortest tLTAg reported so far is only 163 aa long [99], and the longest tLTAg variant reported is 538 aa long [accession number KJ022619]. Deletion always removes the C-terminal DNA-binding, ATPase/helicase and the putative p53-binding domains but retains the N-terminal DnaJ motif that interacts with HSc70 and the pRb-binding domain [70,92,100,101]. LTAg does not appear to interact directly with p53 but may have an indirect effect through direct binding of the ubiquitin-specific protease 7 (Usp7), which controls p53 stability [101]. The mechanisms not involving the DDR by which LTAg and sTAg may contribute to oncogenesis are beyond the scope of this review and have been extensively discussed by others [57,89,102,103,104].

## 4. Merkel Cell Polyomavirus and DDR

### 4.1. BKPyV and JCPyV and the ATM and ATR DDR

Viruses use the host cell replication machinery for the replication of their genomes. Viruses usurp the DDR and either use it to efficiently replicate their genomes or abuse it, leading to host genomic instability and ultimately contributing to oncogenesis [105,106,107,108]. The human tumor viruses, human papillomaviruses (HPV), Epstein–Barr virus (EBV or human herpesvirus 4), Kaposi’s sarcoma-associated herpesvirus (KSHV or human herpesvirus 8), hepatitis B virus (HBV), human T-lymphotropic virus type 1 (HTLV-I), and hepatitis C virus (HCV) all interfere with the accuracy of the DDR pathways, thereby predisposing infected cells to genomic instability (for reviews, see [105,106,108,109,110,111,112]). In the next paragraph, we briefly summarize findings of the interaction between BKPyV and JCPyV and the ATM and ATR pathways. Readers are referred to excellent reviews and original references for details.

Studies of BKPyV, JCPyV and MCPyV have shown that infection with these viruses modulates the ATM and ATR pathways. These viruses hijack proteins from the DDR pathways to favor their DNA replication, but, at the same time, this disadvantages the host cell’s genomic stability, potentially contributing to tumorigenesis [108,113,114]. Infection with JCPyV or expression of the viral LTAg and sTAg has been shown to cause genomic instability and DNA damage in numerous cell lines; trigger phosphorylation of ATM, ATR, CHK1, CHK2 and H2AX; and increase the levels of DNA repair proteins. Moreover, DNA repair enzymes co-localized with the replication sites of JCPyV DNA. Agnoprotein, a viral protein expressed by BKPyV and JCPyV, but not MCPyV, was reported to bind and interfere with the functions of p53 and Ku70, a protein involved in the repair of DSBs [113,115,116]. BKPyV infection of renal proximal tubule epithelial cells resulted in increased phosphorylation of ATM, ATR, CHK1, CHK2, and γH2AX, as well as p53 levels. Interestingly, double knockdown of ATM and ATR did not abrogate BKPyV-induced H2AX phosphorylation [117]. BKPyV-triggered phosphorylation of DDR proteins probably occurs through the sTAg-mediated inhibition of protein phosphatase 2A [118]. Manipulating the ATM and ATR pathways optimized BKPyV DNA replication but can compromise host genome integrity [117]. The BKPyV agnoprotein interacts with the proliferating cell nuclear antigen (PCNA), possibly affecting its role in DNA repair [119].

### 4.2. MCPyV and DDR

#### 4.2.1. MCC and Mutation in DDR Genes

Mutations in DDR genes have been reported in both virus-negative and virus-positive MCC specimens. Exome sequencing of 263 genes in 19 MCC samples (9 virus positive and 10 virus negative) identified three patients who were virus positive and three who were virus negative with mutations in one or several DDR genes. Four samples (of which three were virus positive) had mutations in the *ATM* gene, five specimens (of which two were virus positive) had a mutated *ATR* gene, and one virus-negative MCC specimen had a mutated *CHEK2* gene. One virus-negative sample had mutations in all three genes, and three virus-positive samples had double mutations: two with mutated *ATM* and *ATR* genes and one with mutations in *ATM* and *CHEK2* genes [120]. A study by Harms et al. detected a frameshift mutation in the ATM gene in one out of 16 MCC samples. The sample was negative for MCPyV, and the deletion resulted in an inactive ATM kinase [121]. Another study on 17 patients with MCC with unknown virus status found one patient with a mutation in the *ATM* gene and another patient with a mutation in the *CHEK2* gene [122]. Investigation of 71 MCC tumors (46 virus positive) resulted in the detection of 16, 15, 11 and 10 mutations in *ATM*, *ATR*, *CHEK1* and *CHEK2* genes, respectively [123]. Although relatively few MCC samples were tested, the available evidence suggests that mutations in DDR genes are unlikely to represent a major driver of MCC oncogenesis, and their functional relevance remains to be established.

#### 4.2.2. MCPyV-Infected Cells and Virus-Positive MCC and the DDR Pathways

Several studies have demonstrated that MCPyV manipulates the DDR in multiple ways, a mechanism that may contribute to tumorigenesis (summarized in Table 2).

The group of You was the first to show that MCPyV infection of the human osteosarcoma cell line U2OS elicited DDR pathways, as shown by higher phosphorylation of the DDR markers γH2AX, ATM, CHK1 and CHK2 compared to non-infected cells [124]. These findings were confirmed in the HPV-negative cervical carcinoma cell line C33A [125]. Expression of LTAg alone provoked phosphorylation of CHK1, but not CHK2. Neither sTAg nor 57kT activated the DDR markers. Experiments with different LT mutants showed that LTAg-induced DNA damage and CHK1 phosphorylation can be assigned to the C-terminal region containing the DNA-binding helicase domain and p53- binding domain, which is deleted in virus-positive MCC [124,126]. LTAg also increased total p53 levels and phosphorylation of p53 at Ser-15, which potentiates p53 transcriptional activity [127]. The N-terminal region seemed dispensable, and LTAg caused p53 phosphorylation through the ATR/CHK1 pathway [124]. One p53 target gene is *CHEK1*. Another is *CDKN1A*, encoding the cyclin-dependent kinase inhibitor p21 [128]. Ectopic expression of both full-length LTAg, but not C-terminal-truncated LTAg, increased p21 mRNA and protein levels. The authors also demonstrated that full-length LTAg caused S-phase cell cycle arrest, whereas a C-truncated variant promoted cell cycle progression into the S phase. Thus, full-length LTAg inhibited cellular proliferation, whereas the truncated variant had the opposite effect. Taken together, these results map the ability of LTAg to inhibit cell proliferation by activating the ATR/CHK1/p53/p21 pathway to the C-terminal region [124]. The mechanism by which MCPyV activates ATM needs to be determined. 

In a follow-up study, this group provided experimental evidence that LTAg, Nbs1 and the phosphorylated forms of H2AX, ATM, ATR, CHK1, CHK2 and RPA32 co-localized at the actively replicating viral DNA complexes in distinct nuclear foci. Chemical inhibitors of ATM and ATR or siRNA-mediated knockdown of these kinases were found capable of reducing MCPyV DNA replication, suggesting that DDR components are required for optimal viral DNA replication [125]. However, the mechanisms by which viral DNA replication benefits from DDR proteins remain unknown. The co-localization of LTAg and ATM is further underscored by in vitro research reporting that ATM can bind and phosphorylate LTAg at Ser-816. ATM-mediated phosphorylation of Ser-816 seems important for the inhibition of cell proliferation by inducing apoptosis but had no effect on viral DNA replication and transcription because the LTAg Ala-816 mutant could not prevent cell proliferation but still supported the replication and transcription of the viral genome [129].

Hyperphosphorylation of histone H2AX in MCC was associated with the presence of MCPyV, and overexpression of MCPyV sTAg in HEK293 cells induced phosphorylation of ATM and its substrates p53 binding protein 1 (TP53BP1) and CHK2 [130]. This observation was not limited to MCPyV sTAg, as overexpression of sTAg from HPyV6, HPyV7 and TSPyV was also induced although with lower stoichiometry and hyperphosphorylation of these DDR proteins. However, the expression levels of the different HPyVs were not compared, nor was the molecular mechanism for sTAg-induced hyperphosphorylation of H2AX, TP53BP1 and CHK2 investigated [130]. Studies with a transgenic mouse model co-expressing MCPyV tLTAg and sTAg revealed increased phosphorylation of ATM and H2AX within the papillomas but not in the surrounding skin despite expression of tLTAg and sTAg in both. This indicates that the cellular environment may contribute to tLTAg+sTAg-induced DDR activation [131]. A transgenic mouse model with epidermal sTAg expression also displayed increased γH2AX levels [132].

Demetriou and co-workers showed that the virus-positive MCC cell line MKL-1 displayed increased sensitivity to DNA damage and reduced levels of the DNA damage recognition protein xeroderma pigmentosa group C (XPC) compared to virus-negative UIOS cells following exposure to UV radiation [133]. Their results indicate that MCPyV-positive cells have diminished DNA repair. The authors then investigated the role of the early proteins. They ectopically expressed wild-type T antigens (i.e., co-expression of LTAg, 57kT and sTAg), mutant T antigens (expressing tLTAg and sTAg, but not 57kT), and LTAg or sTAg alone in UIOS cells. Expression of mutant T antigens, but not LTAg, sTAg or the wild-type T antigens, resulted in loss of repair of UV-induced DNA damage. However, both mutant and wild-type T antigens reduced the protein levels of XPC, p53 and p21 [133]. Similarly, mutant T antigens, but not wild-type T antigens, inhibited DNA repair and reduced XPC levels when ectopically expressed in normal human dermal fibroblasts [133]. These findings suggest that tLTAg, rather than full-length LTAg and sTAg, interferes with DNA damage repair and therefore may contribute to the oncogenesis of virus-positive MCC.

An elegant study employing the MCPyV-positive MCC cell line WaGa revealed that the expression of tLTAg is allied with the augmented expression of DDR proteins [134]. The authors used short hairpin RNA to target all MCPyV early mRNAs. Repression of early protein expression resulted in the >2-fold downregulation of 110 proteins and upregulation of 40 proteins compared to control cells. Of the 40 proteins with higher protein levels, 25 are involved in DDR. The authors then investigated the role of tLTAg in DDR by identifying proximal interactors. These included 46 DDR proteins, such as H2AX, XRCC4, and TP53BP1. Another prominent tLTAg partner was euchromatic histone lysine methyltransferase 2 (EHMT2). This epigenetic modifier of chromatin also plays a role in DDR. EHMT2 has a protective function in replication stress-induced DNA damage by trimethylation of H3K9, and EHMT2-mediated methylation of p53 prevents activation of inactive p53 [135,136]. In WaGa cells, depletion of the early proteins or inhibition of EHMT2 reduced the phosphorylation levels of pATR, pCHK1, pCHK2, and γH2AX, but not pATM. However, shRNA treatment of MKL-1 and PeTa, two other virus-positive MCC cell lines, did not affect γH2AX and pCHK1 levels in MKL-1, while pCHK1, but not γH2AX levels, were reduced in PeTa cells. Inhibition of EHMT2 resulted in increased levels of pCHK1 in both virus-positive and virus-negative MCC cell lines. Downregulation of t antigens decreased cell proliferation compared with cells expressing t antigens. Cell growth was rescued by inhibition of EHMT2. In conclusion, tLTAg and EHMT2 cooperate to regulate the DDR pathway. The intimate association between tLTAg and EHMT2, in which tLTAg acts as a scaffold enhancing the interaction between EHMT2 and DDR proteins [134], could explain the relative genetic stability and continuous growth of MCC [81,137].

In a pilot study on seven MCC samples, three of which were virus positive, our group found significantly increased mRNA levels of the *ATM*, *ATR*, *CHEK1*, and *CHEK2* genes in MCPyV-positive specimens compared to virus-negative ones [138]. These results were confirmed in a subsequent study including 19 MCC specimens, 11 of which were virus positive [139]. Transcript levels of *H2AFX*, *TP53*, *RAD51*, and *CDKN1A* genes were also significantly increased in virus-positive compared with virus-negative tumors [139].

Bhatia and colleagues examined p53 protein expression in nine MCC samples with high viral abundance (≥1 genome copy/16 cells), eight samples with low viral abundance (<1 copy/300 cells) and six virus-negative specimens [140]. Although p53 was detectable in all tumors with high viral abundance, a high viral copy number correlated with lower p53 levels compared to samples with a low copy number or no virus. This appears to be in contrast with our findings reporting elevated *p53* mRNA levels in virus-positive samples [138,139]. However, this comparison should be interpreted with caution, as the two studies employed different detection methods (immunohistochemistry versus reverse transcriptase-polymerase chain reaction). In addition, differences in viral-load quantification between the studies, together with the relatively small number of specimens and the overlap in p53 expression between some groups in the study by Bhatia et al., further limit comparisons. Overall, these differences make a clear relationship between MCPyV status, viral abundance, and p53 levels difficult to establish.

**Table 2:** Effect of MCPyV on the DDR pathway.

Viral Component	DDR Component	Sample	Reference
Integrated viral genome	mutation in *ATM*, *ATR*, *CHEK1*, *CHEK2* genes	VP-MCC ^#^	[120,122,123]
Virions	increased phosphorylation of H2AX, ATM, CHK1, CHK2	U2OS and C33A cells	[124,125]
LTAg	increased phosphorylation of CHK1, p53	U2OS	[125]
Integrated viral genome	increased phosphorylation of H2AX	VP-MCC ^#^	[130]
sTAg	increased phosphorylation of ATM, CHK2	HEK293	[130]
sTAg+tLTAg	Increased phosphorylation of H2AX, ATM	transgenic mouse	[131]
sTAg	increased phosphorylation of H2AX	transgenic mouse	[132]
LTAg+sTAg+57 kT	reduced protein levels of p53, p21, XPC	UIOS	[133]
tLT+sT	reduced protein levels of p53, p21, XPC	UIOS	[133]
	reduced protein levels of XPC	hDF *	
KD ^‡^ of LTAg+sTAg+57 kT	increase levels of 25 DDR proteins, including CHEK1 and PCNA	WaGa	[134]
Integrated viral genome	increased mRNA levels of *ATM*, *ATR*, *CHK1*, *CHK2*, *H2AX*, *p53*, *RAD51*, *p21*	VP-MCC ^#^	[138,139]
Integrated viral genome	reduced p53 protein levels	VP-MCC ^#^	[140]
Virions	increased mRNA levels of *ATM*, *ATR*, *CHK1*, *CHK2*, *H2AX*, *p53*, *RAD51*, *p21*	A549, H1299, HBEC-KT, ALI	[141]
Integrated viral genome	increased miR-182 levels; possible target for CHK2	MS1	[142]

^#^ VP = virus positive MCC; * human dermal fibroblast; ^‡^ shRNA-mediated knockdown.

#### 4.2.3. The DDR Pathway in MCPyV-Infected Cells of the Respiratory Tract

While the natural host cells for MCPyV are dermal fibroblasts, cells from the respiratory tract may represent the primary target cell upon infection [143].

Our group confirmed enhanced transcription levels of DDR genes upon viral infection of the lung cancer cell lines A549 and H1299 and non-malignant bronchial HBEC-KT and ALI models [141]. All four cell models were permissive for viral infection and produced infectious virus particles upon transfection of MCPyV DNA. Compared to non-infected cells, transcription levels of the *ATM*, *ATR*, *CHEK1*, *CHEK2*, *H2AFX*, *RAD51*, *TP53* and *CDKN1A* genes were all elevated at 2 and 4 days after infection in all four cell systems, while they remained upregulated at 8 days in HBEC-KT and ALI, except for *RAD51* in HBEC-KT [141]. These results suggest that MCPyV can manipulate the mRNA levels of genes encoding proteins involved in the DDR in cell models of the respiratory tract. Hence, the virus exploits the DDR to create a cellular environment that favors viral replication [125]. Furthermore, these findings support the assumption that cells from the respiratory tract may be the primary target of MCPyV infection and underscore a possible link between MCPyV and lung malignancies [144,145,146,147].

#### 4.2.4. MicroRNA and DDR 

MicroRNAs (miRNAs) can also control the expression levels of DDR proteins. DNA damage triggers the expression of miRNAs that have been shown to modulate levels of several DDR proteins (reviewed in [148]). Upregulation of miR-18a-5p and miR-421 downregulated ATM levels, while increased concentrations of miR-15b enhanced pATM levels by inhibiting the protein phosphatase WIP1. miR-185 and -383 depleted ATR levels, whereas miR-16, -26a, -195 and -495 knocked down CHK1 protein levels. CHK2 expression was repressed by miR-182-5p. Upregulation of miR-15b and miR-494 resulted in increased γH2AX levels [148]. Of the miRNAs that target DDR mRNAs, miR-182 was upregulated in MCC cells compared to normal skin cells, and levels were high in MCPyV-positive MS1 MCC cells [142]. The biological significance that may link miR-182 to CHK2 in virus-positive MCC needs to be elaborated.

## 5. Therapeutic Strategies Targeting DDR in Merkel Cell Polyomavirus-Positive Merkel Cell Carcinoma

Standard MCC treatment consists of surgery, radiation and chemotherapy [78,79,149]. In recent years, immunotherapy with the checkpoint inhibitors anti-PD-1 or anti-PD-L1 has been applied with improved outcomes in about 50% of patients [150,151]. Notably, to date, treatment with these inhibitors is standard for advanced disease where complete resection is not feasible [152]. Despite partial success, novel therapeutic strategies to increase the antitumor effect are needed. The link between MCPyV and the DDR makes the DDR an attractive target in virus-positive MCC. Indeed, DDR inhibitors are currently used in cancer therapy [153].

The cytotoxic effects of the ATR inhibitor ceralasertib (or AZD6738 [154]), the CHK1 inhibitor prexasertib (or LY2606368 [155]) and the nucleoside analog gemcitabine (or 2’,2’-difluorodeoxycytidine, [156]), which pauses DNA replication and induces DNA damage, alone or in combination were tested on virus-negative and virus-positive MCC cell lines [157]. Gemcitabine alone had little cytotoxic effect on virus-positive MCC cells, whereas combination with either the ATR inhibitor or the CHK1 inhibitor resulted in increased apoptosis compared to untreated cells. Combinational treatment of MCPyV-negative MCC cell lines had no significant cytotoxic effect. These results indicate that virus-positive MCC cell lines are more sensitive to combined treatment with ATR/CHK1 inhibitors and gemcitabine than virus-negative cell lines and may therefore be used in patients with MCC with MCPyV-positive tumors. A recent study on pancreatic ductal adenocarcinoma reported that high levels of CD44 reduce sensitivity to gemcitabine [158]. CD44 expression levels may correlate with the risk of metastasis in patients with MCC, as no CD44 expression on primary or recurrent MCC tumors (14 out of 19 specimens) was observed, whereas 3 out of 6 metastatic MCC tumors had CD44 expression [159]. This warrants screening for CD44 expression in patients with MCC status prior to therapy involving gemcitabine to increase a positive response to the treatment. A clinical trial with patients with MCC and the ATR inhibitor M1774 or tuvusertib is ongoing (clinicaltrials.gov ID number NCT05947500). Selinexor (KPT-330), a selective inhibitor of nuclear exportin 1, was shown to downregulate the expression of LTAg and sTAg in MCPyV-positive cell lines [160]. This inhibitor also inhibits cell proliferation and downregulates the protein levels of ATR, ATM, CHK1 and CHK2 in virus-negative and virus-positive MCC cell lines [161] but so far has not been tested in MCC therapy. Other ATR inhibitors include elimusertib, camonsertib, berzosertib, ART0380, and gartisertib, but they have not been studied in the context of MCC (clinicaltrials.gov, accessed in September 2026 and [162]). 

Specific ATM inhibitors, including toxasertib (AZD0156), M3541, M4076 and others, have shown antioncogenic potential in vitro, and although some have entered clinical studies in, e.g., patients with solid tumors, patients with MCC have not been enrolled [163,164,165,166] (clinicaltrials.gov, accessed in September 2026). 

Next-generation specific DNA-PK inhibitors—such as peposertib (M3814), AZD7648, and nedisertib—and CHK1 and CHK2 inhibitors have been developed, but their usefulness in MCC therapy remains to be tested [167,168] (clinicaltrials.gov, accessed in September 2026).

Interestingly, a synergism was observed between checkpoint inhibitors and inhibitors of the DDR in pre-clinical studies (for an excellent review, see [169]). Since patients with MCC are unresponsive or become refractory to checkpoint inhibitor immunotherapy, combination with DDR inhibitors could be helpful for these patients. A clinical phase II study compares the effect of ATR inhibitor M1774 (tuvusertib) alone or in combination with PD-L1 inhibitor avelumab in patients with MCC (clinicaltrials.gov, ID number NCT05947500).

Low concentrations (IC_50_ = 150 nM) of the phytochemical glaucarubin, identified by screening a natural library, efficiently kill virus-positive MCC cell lines but were well-tolerated at high concentrations (IC_50_ ranges from 5–150 μM) by virus-negative cell lines and human dermal fibroblasts [170]. Glaucarubin treatment resulted in accumulation of DSBs and accumulation of γH2AX and phospho-Ser15 p53 levels in the virus-positive cell lines but not in the other cells. Combined with the FDA-approved BCL-2 inhibitor ABT-199 (or venetoclax), complete cell death was obtained [170]. One clinical study of patients with MCC and venetoclax is ongoing (clinicaltrials.gov, ID number NCT03000257), but the use of glaucarubin in clinical trials has not been reported.

## 6. Future Perspectives

The viral proteins LTAg (either full length or truncated) and sTAg are involved in the interaction with the DDR pathway, but the exact mechanisms remain elusive. Further studies are required to unravel the molecular mechanisms by which these viral proteins interact with DDR pathways. Mapping the functional domains on the LTAg and sTAg required for MCPyV-induced phosphorylation of DDR proteins may provide important clues to the effect of MCPyV on the DDR.

The interaction between MCPyV and the DDR was investigated in MCC cell lines, MCC tumors and in MCPyV-infected cells. For the latter, U2OS, HEK293 and cells of the respiratory tract were used (see Section 4.2.3). Similar studies in human dermal fibroblasts, as these are natural host cells [171], are relevant but are lacking. Infection of cells with MCPyV and virus-positive MCC cells display increased phosphorylation levels of the DDR proteins ATM, ATR, CHK1, CHK2 and H2AX, whereas our studies demonstrated that MCPyV can also increase the transcript levels of DDR genes. These findings suggest that the interaction of MCPyV with the DDR pathway components is at two levels: at the transcriptional level and at the post-translational level. 

Enhanced transcript levels of DDR proteins in MCPyV-infected cells may be the result of the transactivating activity of LTAg and sTAg on heterologous promoters. LTAg of other polyomaviruses not only regulates transcription from its own promoter but can also affect transcription from cellular promoters [172,173]. It is thus plausible that MCPyV LTAg increases mRNA in DDR genes through transactivation of their promoters. MCPyV sTAg can also activate transcription from specific promoters through its interaction with the MycL-EP400 complex [96]. The authors identified 2157 differentially expressed genes when comparing MKL-1 cells (i.e., a MCPyV-positive MCC cell line) with MKL-1 cells in which MycL and EP400 had been depleted by short hairpin RNA treatment. Hence, sTAg may stimulate transcription of DDR genes in a MycL: EP400-dependent manner. Another mechanism for regulating transcriptional levels of DDR components could involve EHMT2. By inhibiting the transcriptional repression function of EHMT2, tLTAg may induce expression of cellular genes (see Section 2). Whether transcription of DDR genes is activated by this mechanism remains unknown. Another scenario could involve pRb [70,92,100]. Like SV40 LTAg, MCPyV LTAg or tLTAg can interact with pRb, thus disrupting the complex pRb: E2F, releasing E2F and resulting in transcription of E2F-responsive genes [174,175]. The DDR gene *CHEK1* is an E2F-responsive gene [176,177,178]. Thus, LTAg and tLTAg may indirectly increase CHEK1 mRNA levels in an E2F-mediated manner through binding pRb and disrupting the pRb: E2F complex.

Increased phosphorylation of DDR proteins by MCPyV could be accomplished by sTAg-mediated inhibition of protein phosphatase 2A (PP2A). Indeed, the sTAg of SV40, BKPyV and JCPyV can interact with and inhibit PP2A, resulting in increased phosphorylation of PP2A target proteins [118,179,180]. MCPyV sTAg also binds PP2A, but it is unknown whether this may affect dephosphorylation of PP2A target proteins [96,181]. MCPyV sTAg has been shown to interact with PP4 and to modulate the phosphorylation of PP4 substrates [182,183]. One of the PP4 substrates is H2AX, suggesting that increased γH2AX levels are the result of the inhibition of PP4 by MCPyV sTAg (reviewed in [184]). 

The early region encodes two other proteins, 57kT and ALTO, whose possible role in the association with the DDR has not been addressed. ALTO expression is downregulated in MCC and may therefore not be implicated in the activation of DDR proteins in tumors [68]. ALTO was shown to activate TBK1. Interestingly, TBK1 can stimulate autophosphorylation of ATM [185]. Thus, in cells expressing ALTO, increased phosphorylation levels of ATM can be partially attributed to the ALTO/TBK pathway.

Another unanswered question is whether the interaction between MCPyV and the DDR is reciprocal. Specifically, it remains to be fully elucidated whether protein kinases of the DDR pathway can phosphorylate and modulate the activity of LTAg and sTAg. As previously discussed, ATM can phosphorylate LTAg at Ser 816. Mass spectrometry studies illustrated that MCPyV is a phosphoprotein and is heavily phosphorylated in the N-terminal part. The putative DNA-PK and ATM phosphorylation sites are Ser-138, -151, -156, -217, -254, and -647, and Thr-809 [186]. Mass spectrometry confirmed phosphorylation of Ser-254 [187] but not of the other sites, and the protein kinases responsible for presumed phosphorylation have also not been identified. Whether these possible modifications of LTAg by DDR kinases have an impact on the functions of LTAg remains elusive. No putative phosphorylation sites for DDR kinases were predicted in sTAg [186].

In conclusion, MCPyV seems to use DDR pathways for opposing purposes. In cells with non-integrated virus expressing full-length LTAg, the virus engages the DDR to create optimal conditions for viral DNA replication and to prevent host DNA damage, which may result in death of the host cell on which the virus completely depends for its life cycle. In virus-positive MCCs, clonal integration of the viral genome and expression of tLTAg are established hallmarks of oncogenesis, whereas the contribution of DDR pathways to tumor development remains to be fully defined.

Given that DDR alterations have been identified in both MCPyV-positive and MCPyV-negative MCCs, their functional relevance may differ depending on viral status. In MCPyV-negative MCC, the UV-associated mutational landscape favors the accumulation of alterations in key tumor suppressors and DDR genes, potentially impairing the cellular response to DNA damage [81]. In MCPyV-positive MCC, which displays a lower mutational burden, DDR dysregulation is likely driven by virus-dependent mechanisms, including transcriptional reprogramming of DDR genes and direct interactions between viral oncoproteins and the DDR factors that modulate their phosphorylation and activity [130,138,139]. In this framework, DDR activity may contribute at different stages. In an early phase, LTAg-driven DDR activation, coupled with perturbation of the host cell cycle, may induce genomic instability, which promotes integration of the viral genome into the host chromosome, an event observed in the majority of MCPyV-positive MCCs [70,76,138,188]. At later stages, tumor cells appear to retain a functional, and in some contexts overstimulated, host DDR, which may enhance tolerance to DNA damage and sustain continuous growth while maintaining relative genomic stability [134,138].

Future studies are needed to elucidate whether DDR dysregulation represents a driver, a co-factor, or a consequence of MCC development.

Clarifying the role of the DDR in MCC oncogenesis, unraveling the molecular mechanisms of the MCPyV-DDR interaction, may allow the design of novel therapeutic strategies for patients with MCPyV-positive MCC.

## Figures and Tables

**Table 1:** Summary of HPyVs assigned by the International Committee on Taxonomy of Viruses. Viral proteins and miRs encoded by their genome and association to human diseases.

Human Polyomaviruses (HPyVs)	Early Proteins	Late Proteins	miR	Human Disease
BK Polyomavirus (BKPyV)	LTAg, sTAg, truncTAg	VP1, VP2, VP3, agnoprotein	yes	PyV-associated nephropathy and hemorrhagic cystitis;
				possibly bladder cancer
JC Polyomavirus (JCPyV)	LTAg, sTAg, T’135, T’136, T’165	VP1, VP2, VP3, agnoprotein	yes	PML ^‡^
				possibly colorectal and brain cancer
Karolinska Institutet polyomavirus (KIPyV)	LTAg, sTAg	VP1, VP2, VP3	NK *	
Washington University polyomavirus (WUPyV)	LTAg, sTAg	VP1, VP2, VP3	NK *	
Merkel cell polyomavirus (MCPyV)	LTAg, sTAg, 57 kT, ALTO	VP1, VP2	yes	MCC
Human Polyomavirus 6 (HPyV6)	LTAg, sTAg	VP1, VP2, VP3	NK *	pruritic rashes and dyskeratotic dermatosis
Human Polyomavirus 7 (HPyV7)	LTAg, sTAg	VP1, VP2, VP3	NK *	pruritic rashes and dyskeratotic dermatosis
Trichodysplasia spinulosa-associated polyomavirus (TSPyV)	LTAg, sTAg, MTAg ^#^, ALTO, 21 kT, Tiny T	VP1, VP2, VP3	NK *	trichodysplasia spinulosa
Human Polyomavirus 9 (HPyV9)	LTAg, sTAg	VP1, VP2, VP3	NK *	
Malawi polyomavirus (MWPyV)	LTAg, sTAg	VP1, VP2, VP3	NK *	
St. Louis polyomavirus (STLPyV)	LTAg, sTAg	VP1, VP2, VP3	NK *	
New Jersey polyomavirus (NJPyV)	LTAg, sTAg	VP1, VP2, VP3	NK *	

^#^ Middle Tag; * NK: not known; ^‡^ progressive multifocal leukencephalopathy.

## Data Availability

No new data were created or analyzed in this study. Data sharing is not applicable to this article.

## Abbreviations

The following abbreviations are used in this manuscript:

ALTO	Alternate frame of Large T Open reading frame
ATM	Ataxia-telangiectasia mutated
ATR	Ataxia-telangiectasia and Rad3-related protein
BER	Base Excision Repair
BKPyV	BK Polyomavirus
CDC25A	Cyclin division cycle 25A
CDK2	Cyclin-dependent kinase 2
*CDKN1A*	Cyclin-dependent kinase inhibitor 1A (gene)
*CHEK1*	Checkpoint kinase 1 (gene)
*CHEK2*	Checkpoint kinase 2 (gene)
CHK1	Checkpoint kinase 1 (protein)
CHK2	Checkpoint kinase 2 (protein)
DDR	DNA damage response
DNA-PK	DNA–protein kinase
ds	Double-stranded
DSB	Double-strand breaks
EBV	Epstein–Barr virus
EHMT2	euchromatic histone lysine methyltransferase 2
*H2AFX*	H2A histone family member X (gene)
H2AX	H2A histone family member X (protein)
HBV	Hepatitis B virus
HCV	Hepatitis C virus
HPV	Human papillomavirus
HPyV	Human polyomavirus
HR	Homologous Recombination
HTLV-I	Human lymphotropic virus type I
JCPyV	JC Polyomavirus
KIPyV	Karolinska Institutet polyomavirus
KSHV	Kaposis’s sarcoma-associated herpes virus
LTAg	Large T antigen
MCC	Merkel cell carcinoma
MCPyV	Merkel cell polyomavirus
MMR	Mismatch Repair
MWPyV	Malawi polyomavirus
NCCR	Non-coding control region
NER	Nucleotide Excision Repair
NHEJ	Non-homologous end joining
NJPyV	New Jersey polyomavirus
p21	Cyclin-dependent kinase inhibitor 1A (protein)
PCNA	Proliferating cell nuclear antigen
PP2A	Protein phosphatase 2A
PyV	Polyomavirus
*RAD51*	Radiation sensitive protein 51 (gene)
RAD51	Radiation sensitive protein 51 (protein)
RPA	Replication protein A
Ser	Serine
ss	Single-stranded
SSB	Single-strand break
SSBR	Single-strand break repair
SV40	Simian virus 40
sTAg	Small t antigen
STING	Stimulator of Interferon Genes
STPyV	St. Louis polyomavirus
TBK1	Tank-binding kinase 1
tLTAg	Truncated Large T antigen
Thr	Threonine
TP53BP1	Tumor protein p53 binding protein 1
TSPyV	Trichodysplasia spinulosa-associated polyomavirus
Usp7	Ubiquitin-specific protease 7
VP	Viral protein
WUPyV	Washington University polyomavirus
XPC	Xeroderma pigmentosa group C
XRCC4	X-ray cross-complementing protein 4

## References

[1] Benedetti F., Curreli S., Gallo R.C., Zella D. (2021). Tampering of Viruses and Bacteria with Host DNA Repair: Implications for Cellular Transformation. Cancers.

[2] Saxena S., Zou L. (2022). Hallmarks of DNA replication stress. Mol. Cell.

[3] Liu N., Du J., Ge J., Liu S.B. (2025). DNA damage-inducing endogenous and exogenous factors and research progress. Nucleosides Nucleotides Nucleic Acids.

[4] Rajković M., Deksne G., Živković L., Leonova E., Spremo-Potparević B., Sjakste N. (2025). DNA damage induced by parasitic infections in humans and animals. Comp. Immunol. Microbiol. Infect. Dis..

[5] Ayna A., Caglayan C., Taysi S. (2025). Cellular and Molecular Mechanisms of Oxidative DNA Damage and Repair. Medicina.

[6] Jackson S.P., Bartek J. (2009). The DNA-damage response in human biology and disease. Nature.

[7] Ciccia A., Elledge S.J. (2010). The DNA damage response: Making it safe to play with knives. Mol. Cell.

[8] Maréchal A., Zou L. (2013). DNA damage sensing by the ATM and ATR kinases. Cold Spring Harb. Perspect. Biol..

[9] Blackford A.N., Jackson S.P. (2017). ATM, ATR, and DNA-PK: The Trinity at the Heart of the DNA Damage Response. Mol. Cell.

[10] Yue X., Bai C., Xie D., Ma T., Zhou P.K. (2020). DNA-PKcs: A Multi-Faceted Player in DNA Damage Response. Front. Genet..

[11] Shiloh Y. (2001). ATM and ATR: Networking cellular responses to DNA damage. Curr. Opin. Genet. Dev..

[12] Kurz E.U., Lees-Miller S.P. (2004). DNA damage-induced activation of ATM and ATM-dependent signaling pathways. DNA Repair.

[13] Bakkenist C.J., Kastan M.B. (2003). DNA damage activates ATM through intermolecular autophosphorylation and dimer dissociation. Nature.

[14] Ward I.M., Wu X., Chen J. (2001). Threonine 68 of Chk2 is phosphorylated at sites of DNA strand breaks. J. Biol. Chem..

[15] Shieh S.Y., Ahn J., Tamai K., Taya Y., Prives C. (2000). The human homologs of checkpoint kinases Chk1 and Cds1 (Chk2) phosphorylate p53 at multiple DNA damage-inducible sites. Genes Dev..

[16] Craig A., Scott M., Burch L., Smith G., Ball K., Hupp T. (2003). Allosteric effects mediate CHK2 phosphorylation of the p53 transactivation domain. EMBO Rep..

[17] Ou Y.H., Chung P.H., Sun T.P., Shieh S.Y. (2005). p53 C-terminal phosphorylation by CHK1 and CHK2 participates in the regulation of DNA-damage-induced C-terminal acetylation. Mol. Biol. Cell.

[18] Cimprich K.A., Cortez D. (2008). ATR: An essential regulator of genome integrity. Nat. Rev. Mol. Cell Biol..

[19] Yazinski S.A., Zou L. (2016). Functions, Regulation, and Therapeutic Implications of the ATR Checkpoint Pathway. Annu. Rev. Genet..

[20] Zhao H., Piwnica-Worms H. (2001). ATR-mediated checkpoint pathways regulate phosphorylation and activation of human Chk1. Mol. Cell Biol..

[21] Riley T., Sontag E., Chen P., Levine A. (2008). Transcriptional control of human p53-regulated genes. Nat. Rev. Mol. Cell Biol..

[22] Laptenko O., Beckerman R., Freulich E., Prives C. (2011). p53 binding to nucleosomes within the p21 promoter in vivo leads to nucleosome loss and transcriptional activation. Proc. Natl. Acad. Sci. USA.

[23] Karimian A., Ahmadi Y., Yousefi B. (2016). Multiple functions of p21 in cell cycle, apoptosis and transcriptional regulation after DNA damage. DNA Repair.

[24] Georgakilas A.G., Martin O.A., Bonner W.M. (2017). p21: A Two-Faced Genome Guardian. Trends Mol. Med..

[25] Ticli G., Cazzalini O., Stivala L.A., Prosperi E. (2022). Revisiting the Function of p21^CDKN1A^ in DNA Repair: The Influence of Protein Interactions and Stability. Int. J. Mol. Sci..

[26] Falck J., Mailand N., Syljuåsen R.G., Bartek J., Lukas J. (2001). The ATM-Chk2-Cdc25A checkpoint pathway guards against radioresistant DNA synthesis. Nature.

[27] Rogakou E.P., Pilch D.R., Orr A.H., Ivanova V.S., Bonner W.M. (1998). DNA double-stranded breaks induce histone H2AX phosphorylation on serine 139. J. Biol. Chem..

[28] Stucki M., Jackson S.P. (2006). gammaH2AX and MDC1: Anchoring the DNA-damage-response machinery to broken chromosomes. DNA Repair.

[29] Podhorecka M., Skladanowski A., Bozko P. (2010). H2AX Phosphorylation: Its Role in DNA Damage Response and Cancer Therapy. J. Nucleic Acids.

[30] Bonner W.M., Redon C.E., Dickey J.S., Nakamura A.J., Sedelnikova O.A., Solier S., Pommier Y. (2008). GammaH2AX and cancer. Nat. Rev. Cancer.

[31] Lieber M.R. (2010). The mechanism of double-strand DNA break repair by the nonhomologous DNA end-joining pathway. Annu. Rev. Biochem..

[32] Roos W.P., Kaina B. (2006). DNA damage-induced cell death by apoptosis. Trends. Mol. Med..

[33] Calvignac-Spencer S., Feltkamp M.C., Daugherty M.D., Moens U., Ramqvist T., Johne R., Ehlers B., Polyomaviridae Study Group of the International Committee on Taxonomy of Viruses (2016). A taxonomy update for the family Polyomaviridae. Arch. Virol..

[34] Moens U., Calvignac-Spencer S., Lauber C., Ramqvist T., Feltkamp M.C.W., Daugherty M.D., Verschoor E.J., Ehlers B. (2017). ICTV Virus Taxonomy Profile: Polyomaviridae. J. Gen. Virol..

[35] Ondov B.D., Starrett G.J., Sappington A., Kostic A., Koren S., Buck C.B., Phillippy A.M. (2019). Mash Screen: High-throughput sequence containment estimation for genome discovery. Genome Biol..

[36] Gedvilaite A., Tryland M., Ulrich R.G., Schneider J., Kurmauskaite V., Moens U., Preugschas H., Calvignac-Spencer S., Ehlers B. (2017). Novel polyomaviruses in shrews (Soricidae) with close similarity to human polyomavirus 12. J. Gen. Virol..

[37] Fahsbender E., Altan E., Estrada M., Seguin M.A., Young P., Leutenegger C.M., Delwart E. (2019). Lyon-IARC Polyomavirus DNA in Feces of Diarrheic Cats. Microbiol. Resour. Announc..

[38] Li Y., Huang H., Lan T., Wang W., Zhang J., Zheng M., Cao L., Sun W., Lu H. (2021). First detection and complete genome analysis of the Lyon IARC polyomavirus in China from samples of diarrheic cats. Virus Genes.

[39] Kamminga S., Van der Meijden E., Pesavento P., Buck C.B., Feltkamp M.C.W. (2023). Serology Identifies LIPyV as a Feline Rather than a Human Polyomavirus. Viruses.

[40] Kean J.M., Rao S., Wang M., Garcea R.L. (2009). Seroepidemiology of human polyomaviruses. PLoS Pathog..

[41] Tolstov Y.L., Pastrana D.V., Feng H., Becker J.C., Jenkins F.J., Moschos S., Chang Y., Buck C.B., Moore P.S. (2009). Human Merkel cell polyomavirus infection II. MCV is a common human infection that can be detected by conformational capsid epitope immunoassays. Int. J. Cancer.

[42] Gossai A., Waterboer T., Nelson H.H., Michel A., Willhauck-Fleckenstein M., Farzan S.F., Hoen A.G., Christensen B.C., Kelsey K.T., Marsit C.J. (2016). Seroepidemiology of Human Polyomaviruses in a US Population. Am. J. Epidemiol..

[43] Gossai A., Waterboer T., Hoen A.G., Farzan S.F., Nelson H.H., Michel A., Willhauck-Fleckenstein M., Christensen B.C., Perry A.E., Pawlita M. (2016). Human polyomaviruses and incidence of cutaneous squamous cell carcinoma in the New Hampshire skin cancer study. Cancer Med..

[44] Karachaliou M., Waterboer T., Casabonne D., Chalkiadaki G., Roumeliotaki T., Michel A., Stiakaki E., Chatzi L., Pawlita M., Kogevinas M. (2016). The Natural History of Human Polyomaviruses and Herpesviruses in Early Life—The Rhea Birth Cohort in Greece. Am. J. Epidemiol..

[45] Karachaliou M., Chatzi L., Roumeliotaki T., Kampouri M., Kyriklaki A., Koutra K., Chalkiadaki G., Michel A., Stiakaki E., Kogevinas M. (2016). Common infections with polyomaviruses and herpesviruses and neuropsychological development at 4 years of age, the Rhea birth cohort in Crete, Greece. J. Child Psychol. Psychiatry.

[46] Kamminga S., Van der Meijden E., Wunderink H.F., Touzé A., Zaaijer H.L., Feltkamp M.C.W. (2018). Development and Evaluation of a Broad Bead-Based Multiplex Immunoassay To Measure IgG Seroreactivity against Human Polyomaviruses. J. Clin. Microbiol..

[47] Antonsson A., Neale R.E., O’Rourke P., Wockner L., Michel A., Pawlita M., Waterboer T., Green A.C. (2018). Prevalence and stability of antibodies to thirteen polyomaviruses and association with cutaneous squamous cell carcinoma: A population-based study. J. Clin. Virol..

[48] Kamminga S., Van der Meijden E., De Brouwer C., Feltkamp M., Zaaijer H. (2019). Prevalence of DNA of fourteen human polyomaviruses determined in blood donors. Transfusion.

[49] Jiang M., Abend J.R., Johnson S.F., Imperiale M.J. (2009). The role of polyomaviruses in human disease. Virology.

[50] Dalianis T., Hirsch H.H. (2013). Human polyomaviruses in disease and cancer. Virology.

[51] Nguyen K.D., Lee E.E., Yue Y., Stork J., Pock L., North J.P., Vandergriff T., Cockerell C., Hosler G.A., Pastrana D.V. (2017). Human polyomavirus 6 and 7 are associated with pruritic and dyskeratotic dermatoses. J. Am. Acad. Dermatol..

[52] Oliveira J.M., Luxo C., Matos A.M. (2026). Human Polyomaviruses of Clinical Relevance: Modes of Transmission and Associated Pathologies. Rev. Med. Virol..

[53] Prado J.C.M., Monezi T.A., Amorim A.T., Lino V., Paladino A., Boccardo E. (2018). Human polyomaviruses and cancer: An overview. Clinics.

[54] Chang Y., Moore P.S. (2012). Merkel cell carcinoma: A virus-induced human cancer. Annu. Rev. Pathol..

[55] Becker J.C., Stang A., DeCaprio J.A., Cerroni L., Lebbé C., Veness M., Nghiem P. (2017). Merkel cell carcinoma. Nat. Rev. Dis. Prim..

[56] Pietropaolo V., Prezioso C., Moens U. (2020). Merkel Cell Polyomavirus and Merkel Cell Carcinoma. Cancers.

[57] DeCaprio J.A. (2021). Molecular Pathogenesis of Merkel Cell Carcinoma. Annu. Rev. Pathol..

[58] Kenan D.J., Mieczkowski P.A., Burger-Calderon R., Singh H.K., Nickeleit V. (2015). The oncogenic potential of BK-polyomavirus is linked to viral integration into the human genome. J. Pathol..

[59] Starrett G.J., Buck C.B. (2019). The case for BK polyomavirus as a cause of bladder cancer. Curr. Opin. Virol..

[60] Zhou Z., Wang Z., Xi L., Song N. (2026). John Cunningham Virus and Colorectal Carcinogenesis: Current Evidence, Molecular Mechanisms, and Unresolved Controversies. Rev. Med. Virol..

[61] (2014). IARC Working Group on the Evaluation of Carcinogenic Risks to Humans. Malaria and some polyomaviruses (SV40, BK, JC, and Markel cell viruses). IARC Monogr. Eval. Carcinog. Risks Hum..

[62] Schowalter R.M., Buck C.B. (2013). The Merkel cell polyomavirus minor capsid protein. PLoS Pathog..

[63] DeCaprio J.A., Garcea R.L. (2013). A cornucopia of human polyomaviruses. Nat. Rev. Microbiol..

[64] Moens U., Krumbholz A., Ehlers B., Zell R., Johne R., Calvignac-Spencer S., Lauber C. (2017). Biology, evolution, and medical importance of polyomaviruses: An update. Infect. Genet. Evol..

[65] Carter J.J., Daugherty M.D., Qi X., Bheda-Malge A., Wipf G.C., Robinson K., Roman A., Malik H.S., Galloway D.A. (2013). Identification of an overprinting gene in Merkel cell polyomavirus provides evolutionary insight into the birth of viral genes. Proc. Natl. Acad. Sci. USA.

[66] Yang R., Lee E.E., Kim J., Choi J.H., Kolitz E., Chen Y., Crewe C., Salisbury N.J.H., Scherer P.E., Cockerell C. (2021). Characterization of ALTO-encoding circular RNAs expressed by Merkel cell polyomavirus and trichodysplasia spinulosa polyomavirus. PLoS Pathog..

[67] Wang R., Senay T.E., Luo T.T., Liu W., Regan J.M., Salisbury N.J.H., Galloway D.A., You J. (2024). Merkel cell polyomavirus protein ALTO modulates TBK1 activity to support persistent infection. PLoS Pathog..

[68] Salisbury N.J.H., Amonkar S., Landazuri Vinueza J., Carter J.J., Roman A., Galloway D.A. (2024). Polyomavirus ALTOs, but not MTs, downregulate viral early gene expression by activating the NF-κB pathway. Proc. Natl. Acad. Sci. USA.

[69] Senay T.E., Li X., Shirhattikar S.G., Luo T.T., You J. (2025). A short intrinsically disordered domain of MCPyV ALTO regulates TBK1 signaling during MCPyV infection. J. Virol..

[70] Shuda M., Feng H., Kwun H.J., Rosen S.T., Gjoerup O., Moore P.S., Chang Y. (2008). T antigen mutations are a human tumor-specific signature for Merkel cell polyomavirus. Proc. Natl. Acad. Sci. USA.

[71] Feng H., Kwun H.J., Liu X., Gjoerup O., Stolz D.B., Chang Y., Moore P.S. (2011). Cellular and viral factors regulating Merkel cell polyomavirus replication. PLoS ONE.

[72] Seo G.J., Chen C.J., Sullivan C.S. (2009). Merkel Cell Polyomavirus Encodes a MicroRNA with the Ability to Autoregulate Viral Gene Expression. Virology.

[73] Lee S., Paulson K.G., Murchison E.P., Afanasiev O.K., Alkan C., Leonard J.H., Byrd D.R., Hannon G.J., Nghiem P. (2011). Identification and validation of a novel mature microRNA encoded by the Merkel cell polyomavirus in human Merkel cell carcinomas. J. Clin. Virol..

[74] Mohsen S.T., Price E.L., Chan A.W., Hanna T.P., Limacher J.J., Nessim C., Shiers J.E., Tron V., Wright F.C., Drucker A.M. (2024). Incidence, mortality and survival of Merkel cell carcinoma: A systematic review of population-based studies. Br. J. Dermatol..

[75] Mistry K., Venables Z.C. (2024). A systematic review of Merkel cell carcinoma epidemiology highlights its rising incidence, poor prognosis and data heterogeneity. Br. J. Dermatol..

[76] Feng H., Shuda M., Chang Y., Moore P.S. (2008). Clonal integration of a polyomavirus in human Merkel cell carcinoma. Science.

[77] Chefitz G., Martin M.R., Vilasi S.M., Saito Y., Jarvis J.E., Hallaert P., Reed D.J., Shalhout S.Z., Brownell I. (2026). Merkel cell carcinoma virus positivity ranges from 57% to 77% in the United States based on geographic region: A pooled cohort study. J. Am. Acad. Dermatol..

[78] Harms P.W., Harms K.L., Moore P.S., DeCaprio J.A., Nghiem P., Wong M.K.K., Brownell I., International Workshop on Merkel Cell Carcinoma Research (IWMCC) Working Group (2018). The biology and treatment of Merkel cell carcinoma: Current understanding and research priorities. Nat. Rev. Clin. Oncol..

[79] Gauci M.L., Aristei C., Becker J.C., Blom A., Bataille V., Dreno B., Del Marmol V., Forsea A.M., Fargnoli M.C., Grob J.J. (2022). Diagnosis and treatment of Merkel cell carcinoma: European consensus-based interdisciplinary guideline—Update 2022. Eur. J. Cancer..

[80] Wong S.Q., Waldeck K., Vergara I.A., Schröder J., Madore J., Wilmott J.S., Colebatch A.J., De Paoli-Iseppi R., Li J., Lupat R. (2015). UV-Associated Mutations Underlie the Etiology of MCV-Negative Merkel Cell Carcinomas. Cancer Res..

[81] Goh G., Walradt T., Markarov V., Blom A., Riaz N., Doumani R., Stafstrom K., Moshiri A., Yelistratova L., Levinsohn J. (2016). Mutational landscape of MCPyV-positive and MCPyV-negative Merkel cell carcinomas with implications for immunotherapy. Oncotarget.

[82] Carter M.D., Gaston D., Huang W.Y., Greer W.L., Pasternak S., Ly T.Y., Walsh N.M. (2018). Genetic profiles of different subsets of Merkel cell carcinoma show links between combined and pure MCPyV-negative tumors. Hum. Pathol..

[83] Vandeven N., Lewis C.W., Makarov V., Riaz N., Paulson K.G., Hippe D., Bestick A., Doumani R., Marx T., Takagishi S. (2018). Merkel Cell Carcinoma Patients Presenting Without a Primary Lesion Have Elevated Markers of Immunity, Higher Tumor Mutation Burden, and Improved Survival. Clin. Cancer Res..

[84] Brazel D., Kumar P., Doan H., Pan T., Shen W., Gao L., Moyers J.T. (2023). Genomic Alterations and Tumor Mutation Burden in Merkel Cell Carcinoma. JAMA Netw. Open.

[85] An P., Sáenz Robles M.T., Pipas J.M. (2012). Large T antigens of polyomaviruses: Amazing molecular machines. Annu. Rev. Microbiol..

[86] Topalis D., Andrei G., Snoeck R. (2013). The large tumor antigen: A “Swiss Army knife” protein possessing the functions required for the polyomavirus life cycle. Antivir. Res..

[87] Shuda M., Kwun H.J., Feng H., Chang Y., Moore P.S. (2011). Human Merkel cell polyomavirus small T antigen is an oncoprotein targeting the 4E-BP1 translation regulator. J. Clin. Investig..

[88] Houben R., Angermeyer S., Haferkamp S., Aue A., Goebeler M., Schrama D., Hesbacher S. (2015). Characterization of functional domains in the Merkel cell polyoma virus Large T antigen. Int. J. Cancer.

[89] Wendzicki J.A., Moore P.S., Chang Y. (2015). Large T and small T antigens of Merkel cell polyomavirus. Curr. Opin. Virol..

[90] Liu W., You J. (2020). Molecular Mechanisms of Merkel Cell Polyomavirus Transformation and Replication. Annu. Rev. Virol..

[91] Liu X., Hein J., Richardson S.C., Basse P.H., Toptan T., Moore P.S., Gjoerup O.V., Chang Y. (2011). Merkel cell polyomavirus large T antigen disrupts lysosome clustering by translocating human Vam6p from the cytoplasm to the nucleus. J. Biol. Chem..

[92] Cheng J., Rozenblatt-Rosen O., Paulson K.G., Nghiem P., DeCaprio J.A. (2013). Merkel cell polyomavirus large T antigen has growth-promoting and inhibitory activities. J. Virol..

[93] Kwun H.J., Shuda M., Feng H., Camacho C.J., Moore P.S., Chang Y. (2013). Merkel cell polyomavirus small T antigen controls viral replication and oncoprotein expression by targeting the cellular ubiquitin ligase SCFFbw7. Cell Host Microbe.

[94] Knight L.M., Stakaityte G., Wood J.J., Abdul-Sada H., Griffiths D.A., Howell G.J., Wheat R., Blair G.E., Steven N.M., Macdonald A. (2015). Merkel cell polyomavirus small T antigen mediates microtubule destabilization to promote cell motility and migration. J. Virol..

[95] Berrios C., Padi M., Keibler M.A., Park D.E., Molla V., Cheng J., Lee S.M., Stephanopoulos G., Quackenbush J., DeCaprio J.A. (2016). Merkel Cell Polyomavirus Small T Antigen Promotes Pro-Glycolytic Metabolic Perturbations Required for Transformation. PLoS Pathog..

[96] Cheng J., Park D.E., Berrios C., White E.A., Arora R., Yoon R., Branigan T., Xiao T., Westerling T., Federation A. (2017). Merkel cell polyomavirus recruits MYCL to the EP400 complex to promote oncogenesis. PLoS Pathog..

[97] Stakaitytė G., Nwogu N., Dobson S.J., Knight L.M., Wasson C.W., Salguero F.J., Blackbourn D.J., Blair G.E., Mankouri J., Macdonald A. (2018). Merkel Cell Polyomavirus Small T Antigen Drives Cell Motility via Rho-GTPase-Induced Filopodium Formation. J. Virol..

[98] Shi H., Yang Y., Gao J., Kumar S., Xie H., Chen Z., Lyu J., Sihto H., Koljonen V., Vega-Rubin-de-Celis S. (2025). Kit-mediated autophagy suppression driven by a viral oncoprotein emerges as a crucial survival mechanism in Merkel cell carcinoma. Autophagy.

[99] Schrama D., Sarosi E.M., Adam C., Ritter C., Kaemmerer U., Klopocki E., König E.M., Utikal J., Becker J.C., Houben R. (2019). Characterization of six Merkel cell polyomavirus-positive Merkel cell carcinoma cell lines: Integration pattern suggest that large T antigen truncating events occur before or during integration. Int. J. Cancer.

[100] Borchert S., Czech-Sioli M., Neumann F., Schmidt C., Wimmer P., Dobner T., Grundhoff A., Fischer N. (2014). High-affinity Rb binding, p53 inhibition, subcellular localization, and transformation by wild-type or tumor-derived shortened Merkel cell polyomavirus large T antigens. J. Virol..

[101] Lim D., Park J.H., Shin H.C., Kim S.J., Ku B. (2026). Molecular analysis of the interaction between ubiquitin-specific protease 7 and large T antigen of Merkel cell polyomavirus. J. Microbiol..

[102] Yang J.F., You J. (2022). Merkel cell polyomavirus and associated Merkel cell carcinoma. Tumour Virus Res..

[103] Ahmed M.M., Cushman C.H., DeCaprio J.A. (2021). Merkel Cell Polyomavirus: Oncogenesis in a Stable Genome. Viruses.

[104] Myrda J., Bremm F., Schaft N., Dörrie J. (2024). The Role of the Large T Antigen in the Molecular Pathogenesis of Merkel Cell Carcinoma. Genes.

[105] Ryan E.L., Hollingworth R., Grand R.J. (2016). Activation of the DNA Damage Response by RNA Viruses. Biomolecules.

[106] Gómez-Moreno A., Garaigorta U. (2017). Hepatitis B Virus and DNA Damage Response: Interactions and Consequences for the Infection. Viruses.

[107] Weitzman M.D., Fradet-Turcotte A. (2018). Virus DNA Replication and the Host DNA Damage Response. Annu. Rev. Virol..

[108] Studstill C.J., Mac M., Moody C.A. (2023). Interplay between the DNA damage response and the life cycle of DNA tumor viruses. Tumour Virus Res..

[109] Hollingworth R., Grand R.J. (2015). Modulation of DNA damage and repair pathways by human tumour viruses. Viruses.

[110] Hau P.M., Tsao S.W. (2017). Epstein-Barr Virus Hijacks DNA Damage Response Transducers to Orchestrate Its Life Cycle. Viruses.

[111] Ohsaki E., Ueda K. (2020). Interplay Between KSHV and the Host DNA Damage Response. Front. Cell. Infect. Microbiol..

[112] Studstill C.J., Moody C.A. (2023). For Better or Worse: Modulation of the Host DNA Damage Response by Human Papillomavirus. Annu. Rev. Virol..

[113] Tahseen D., Rady P.L., Tyring S.K. (2020). Human polyomavirus modulation of the host DNA damage response. Virus Genes.

[114] Collins C.D., Stefely M., Mehta K.P.M., Spurgeon M.E. (2026). Viruses and the host replisome: Discovering oncogenic mechanisms of small DNA tumor viruses. J. Virol..

[115] Darbinyan A., Siddiqui K.M., Slonina D., Darbinian N., Amini S., White M.K., Khalili K. (2004). Role of JC virus agnoprotein in DNA repair. J. Virol..

[116] Moens U., Macdonald A. (2019). Effect of the Large and Small T-Antigens of Human Polyomaviruses on Signaling Pathways. Int. J. Mol. Sci..

[117] Jiang M., Zhao L., Gamez M., Imperiale M.J. (2012). Roles of ATM and ATR-mediated DNA damage responses during lytic BK polyomavirus infection. PLoS Pathog..

[118] Zou W., Imperiale M.J. (2023). Regulation of Virus Replication by BK Polyomavirus Small T Antigen. J. Virol..

[119] Gerits N., Johannessen M., Tümmler C., Walquist M., Kostenko S., Snapkov I., van Loon B., Ferrari E., Hübscher U., Moens U. (2015). Agnoprotein of polyomavirus BK interacts with proliferating cell nuclear antigen and inhibits DNA replication. Virol. J..

[120] Ferrarotto R., Cardnell R., Su S., Diao L., Eterovic A.K., Prieto V., Morrisson W.H., Wang J., Kies M.S., Glisson B.S. (2018). Poly ADP-ribose polymerase-1 as a potential therapeutic target in Merkel cell carcinoma. Head Neck.

[121] Harms P.W., Vats P., Verhaegen M.E., Robinson D.R., Wu Y.M., Dhanasekaran S.M., Palanisamy N., Siddiqui J., Cao X., Su F. (2015). The Distinctive Mutational Spectra of Polyomavirus-Negative Merkel Cell Carcinoma. Cancer Res..

[122] Cohen P.R., Tomson B.N., Elkin S.K., Marchlik E., Carter J.L., Kurzrock R. (2016). Genomic portfolio of Merkel cell carcinoma as determined by comprehensive genomic profiling: Implications for targeted therapeutics. Oncotarget.

[123] Starrett G.J., Thakuria M., Chen T., Marcelus C., Cheng J., Nomburg J., Thorner A.R., Slevin M.K., Powers W., Burns R.T. (2020). Clinical and molecular characterization of virus-positive and virus-negative Merkel cell carcinoma. Genome Med..

[124] Li J., Wang X., Diaz J., Tsang S.H., Buck C.B., You J. (2013). Merkel cell polyomavirus large T antigen disrupts host genomic integrity and inhibits cellular proliferation. J. Virol..

[125] Tsang S.H., Wang X., Li J., Buck C.B., You J. (2014). Host DNA damage response factors localize to merkel cell polyomavirus DNA replication sites to support efficient viral DNA replication. J. Virol..

[126] Moens U., Prezioso C., Pietropaolo V. (2022). Functional Domains of the Early Proteins and Experimental and Epidemiological Studies Suggest a Role for the Novel Human Polyomaviruses in Cancer. Front. Microbiol..

[127] Loughery J., Cox M., Smith L.M., Meek D.W. (2014). Critical role for p53-serine 15 phosphorylation in stimulating transactivation at p53-responsive promoters. Nucleic Acids Res..

[128] Engeland K. (2022). Cell cycle regulation: P53-p21-RB signaling. Cell Death Differ..

[129] Li J., Diaz J., Wang X., Tsang S.H., You J. (2015). Phosphorylation of Merkel cell polyomavirus large tumor antigen at serine 816 by ATM kinase induces apoptosis in host cells. J. Biol. Chem..

[130] Wu J.H., Narayanan D., Limmer A.L., Simonette R.A., Rady P.L., Tyring S.K. (2019). Merkel Cell Polyomavirus Small T Antigen Induces DNA Damage Response. Intervirology.

[131] Spurgeon M.E., Cheng J., Bronson R.T., Lambert P.F., DeCaprio J.A. (2015). Tumorigenic activity of merkel cell polyomavirus T antigens expressed in the stratified epithelium of mice. Cancer Res..

[132] Verhaegen M.E., Mangelberger D., Harms P.W., Vozheiko T.D., Weick J.W., Wilbert D.M., Saunders T.L., Ermilov A.N., Bichakjian C.K., Johnson T.M. (2015). Merkel cell polyomavirus small T antigen is oncogenic in transgenic mice. J. Investig. Dermatol..

[133] Demetriou S.K., Ona-Vu K., Sullivan E.M., Dong T.K., Hsu S.W., Oh D.H. (2012). Defective DNA repair and cell cycle arrest in cells expressing Merkel cell polyomavirus T antigen. Int. J. Cancer.

[134] Bachiri K., Kantar D., Laurent E.M.N., Gaboriaud P., Durand L., Drouin A., Chollot M., Schrama D., Houben R., Kervarrec T. (2025). DNA Damage Stress Control Is a Truncated Large T Antigen and Euchromatic Histone Lysine Methyltransferase 2-Dependent Central Feature of Merkel Cell Carcinoma. J. Investig. Dermatol..

[135] Huang J., Dorsey J., Chuikov S., Zhang X., Jenuwein T., Reinberg D., Berger S.L. (2010). G9a and Glp methylate lysine 373 in the tumor suppressor p53. J. Biol. Chem..

[136] Gaggioli V., Lo C.S.Y., Reverón-Gómez N., Jasencakova Z., Domenech H., Nguyen H., Sidoli S., Tvardovskiy A., Uruci S., Slotman J.A. (2023). Dynamic de novo heterochromatin assembly and disassembly at replication forks ensures fork stability. Nat. Cell Biol..

[137] Horny K., Gerhardt P., Hebel-Cherouny A., Wülbeck C., Utikal J., Becker J.C. (2021). Mutational Landscape of Virus- and UV-Associated Merkel Cell Carcinoma Cell Lines Is Comparable to Tumor Tissue. Cancers.

[138] Passerini S., Fracella M., Ferlosio A., Moens U., Scagnolari C., Antonelli G., Ciotti M., Pietropaolo V. (2025). Investigation of mRNA expression levels of DNA damage response genes in Merkel Cell Polyomavirus-positive Merkel Cell Carcinoma: A pilot study. Discov. Oncol..

[139] Messina S., Mallardo D., Ferlosio A., Festino L., Trojaniello C., Di Trolio R., Ciotti M., Ascierto P.A., Pietropaolo V., Passerini S. (2026). Merkel Cell Polyomavirus and DNA Damage Response (DDR): Transcriptional Analysis of DDR Pathways in the Context of Merkel Cell Carcinoma. Cancers.

[140] Bhatia K., Goedert J.J., Modali R., Preiss L., Ayers L.W. (2010). Merkel cell carcinoma subgroups by Merkel cell polyomavirus DNA relative abundance and oncogene expression. Int. J. Cancer..

[141] Passerini S., De Angelis M., Messina S., Scribano D., Ambrosi C., Moens U., Nencioni L., Pietropaolo V. (2026). Respiratory Models Reveal DNA Damage Response Modulation by Merkel Cell Polyomavirus. Int. J. Mol. Sci..

[142] Ning M.S., Kim A.S., Prasad N., Levy S.E., Zhang H., Andl T. (2014). Characterization of the Merkel Cell Carcinoma miRNome. J. Skin. Cancer.

[143] Mazziotta C., Rotondo J.C. (2025). Emerging in vitro models to study Merkel cell carcinoma pathobiology. Front. Cell Dev. Biol..

[144] Hashida Y., Imajoh M., Nemoto Y., Kamioka M., Taniguchi A., Taguchi T., Kume M., Orihashi K., Daibata M. (2013). Detection of Merkel cell polyomavirus with a tumour-specific signature in non-small cell lung cancer. Br. J. Cancer.

[145] Kim G.J., Lee J.H., Lee D.H. (2017). Clinical and prognostic significance of Merkel cell polyomavirus in nonsmall cell lung cancer. Medicine.

[146] Behdarvand A., Zamani M.S., Sadeghi F., Yahyapour Y., Vaziri F., Jamnani F.R., Nowruzi B., Fateh A., Siadat S.D. (2017). Evaluation of Merkel cell polyomavirus in non-small cell lung cancer and adjacent normal cells. Microb. Pathog..

[147] Zhou C., Lu L., Hu M., Suzuki T., Zhou X. (2025). Merkel cell polyomavirus in lung carcinogenesis: Evidence and controversy. Front. Oncol..

[148] Visser H., Thomas A.D. (2021). MicroRNAs and the DNA damage response: How is cell fate determined?. DNA Repair.

[149] Schadendorf D., Lebbé C., Zur Hausen A., Avril M.F., Hariharan S., Bharmal M., Becker J.C. (2017). Merkel cell carcinoma: Epidemiology, prognosis, therapy and unmet medical needs. Eur. J. Cancer.

[150] Colunga A., Pulliam T., Nghiem P. (2018). Merkel Cell Carcinoma in the Age of Immunotherapy: Facts and Hopes. Clin. Cancer Res..

[151] de Moraes F.C.A., Kreuz M., de Lara I.C.A., Lôbo A.O.M., Burbano R.M.R. (2024). Efficacy and safety of PD-1/PD-L1 inhibitors in patients with Merkel Cell Carcinoma: A systematic review and Meta-analysis. BMC Cancer.

[152] Pan K., Vromans A.M., Cheng L., Grant-Kels J.M., Katz S.C., Hadfield M.J. (2025). Merkel Cell Carcinoma: Current Treatment Landscape and Emerging Therapeutic Targets. Curr. Oncol. Rep..

[153] Groelly F.J., Fawkes M., Dagg R.A., Blackford A.N., Tarsounas M. (2023). Targeting DNA damage response pathways in cancer. Nat. Rev. Cancer.

[154] Foote K.M., Nissink J.W.M., McGuire T., Turner P., Guichard S., Yates J.W.T., Lau A., Blades K., Heathcote D., Odedra R. (2018). Discovery and Characterization of AZD6738, a Potent Inhibitor of Ataxia Telangiectasia Mutated and Rad3 Related (ATR) Kinase with Application as an Anticancer Agent. J. Med. Chem..

[155] King C., Diaz H.B., McNeely S., Barnard D., Dempsey J., Blosser W., Beckmann R., Barda D., Marshall M.S. (2015). LY2606368 Causes Replication Catastrophe and Antitumor Effects through CHK1-Dependent Mechanisms. Mol. Cancer Ther..

[156] Heinemann V., Hertel L.W., Grindey G.B., Plunkett W. (1988). Comparison of the cellular pharmacokinetics and toxicity of 2′,2′-difluorodeoxycytidine and 1-beta-D-arabinofuranosylcytosine. Cancer Res..

[157] Proctor E.V., Elsawa S.F., Walker S.R., Cheng J. (2026). Inhibition of the ATR-CHK1 signaling pathway sensitizes MCPyV-positive Merkel cell carcinoma to gemcitabine. BMC Cancer.

[158] Karabiber A., Zhou Y., Mittelstädt A., Hansen F.J., Litau M., Kuchenreuther I., Mazurie J., Clausen F.N., Klöckner S., Czubayko F. (2026). Immune Cell-Specific and Isoform-Selective Regulation of CD44 in Pancreatic Ductal Adenocarcinoma Links Lymph Node Variant Loss and Exosomal CD44 to Clinical Outcome in Pancreatic Ductal Adenocarcinoma. Cells.

[159] Penneys N.S., Shapiro S. (1994). CD44 expression in Merkel cell carcinoma may correlate with risk of metastasis. J. Cutan. Pathol..

[160] Gupta R., Simonette R.A., Doan H.Q., Rady P.L., Tyring S.K. (2021). Targeting the expression of T antigens with selinexor (KPT-330) shows promise for Merkel cell polyomavirus-positive Merkel cell carcinoma treatment. Br. J. Dermatol..

[161] Moore S.A., Narayanan D., Simonette R.A., Bartley B.R., Doan H.Q., Rady P.L., Tyring S.K. (2022). Selinexor is a novel inhibitor of DNA damage response in Merkel cell carcinoma. Clin. Exp. Dermatol..

[162] Singh G., Sharma R., Gautam V., Dhingra A.K., Hsu S.P., Nepali K. (2026). Recent advances in small molecule ATR kinase inhibitors as anticancer agents. Future Med. Chem..

[163] Lavin M.F., Yeo A.J. (2020). Clinical potential of ATM inhibitors. Mutat. Res..

[164] Waqar S.N., Robinson C., Olszanski A.J., Spira A., Hackmaster M., Lucas L., Sponton L., Jin H., Hering U., Cronier D. (2022). Phase I Trial of ATM Inhibitor M3541 in Combination with Palliative Radiotherapy in Patients with Solid Tumors. Investig. New Drugs.

[165] Du S., Liang Q., Shi J. (2024). Progress of ATM inhibitors: Opportunities and challenges. Eur. J. Med. Chem..

[166] Al-Zoubi R.M., Garada K., Al Huneidi R., Baddar Z.M.H., Zarour A.A., Elaarag M., Al-Zoubi S.R., Al-Qudimat A.R., Al Zoubi M.S., Agouni A. (2025). ATM inhibitors in cancer radiotherapy: Mechanisms, clinical development, and future directions. Eur. J. Med. Chem..

[167] Ronco C., Martin A.R., Demange L., Benhida R. (2016). ATM, ATR, CHK1, CHK2 and WEE1 inhibitors in cancer and cancer stem cells. MedChemComm.

[168] Watson E., Hutchinson J.R.R., Harnor S.J., Gaughan L., Cano C. (2026). Targeting DNA-PK: Medicinal chemistry insights into small-molecule inhibitor discovery and optimisation. RSC Med. Chem..

[169] Goff P.H., Bhakuni R., Pulliam T., Lee J.H., Hall E.T., Nghiem P. (2021). Intersection of Two Checkpoints: Could Inhibiting the DNA Damage Response Checkpoint Rescue Immune Checkpoint-Refractory Cancer?. Cancers.

[170] Liu W., Krump N.A., Herlyn M., You J. (2020). Combining DNA Damage Induction with BCL-2 Inhibition to Enhance Merkel Cell Carcinoma Cytotoxicity. Biology.

[171] Liu W., Yang R., Payne A.S., Schowalter R.M., Spurgeon M.E., Lambert P.F., Xu X., Buck C.B., You J. (2016). Identifying the Target Cells and Mechanisms of Merkel Cell Polyomavirus Infection. Cell Host Microbe.

[172] Pannuti A., Pascucci A., La Mantia G., Fisher-Fantuzzi L., Vesco C., Lania L. (1987). trans-activation of cellular and viral promoters by a transforming nonkaryophilic simian virus 40 large T antigen. J. Virol..

[173] Moens U., Seternes O.M., Johansen B., Rekvig O.P. (1997). Mechanisms of transcriptional regulation of cellular genes by SV40 large T- and small T-antigens. Virus Genes.

[174] Sullivan C.S., Cantalupo P., Pipas J.M. (2000). The molecular chaperone activity of simian virus 40 large T antigen is required to disrupt Rb-E2F family complexes by an ATP-dependent mechanism. Mol. Cell. Biol..

[175] Lee C., Cho Y. (2002). Interactions of SV40 large T antigen and other viral proteins with retinoblastoma tumour suppressor. Rev. Med. Virol..

[176] Carrassa L., Broggini M., Vikhanskaya F., Damia G. (2003). Characterization of the 5′flanking region of the human Chk1 gene: Identification of E2F1 functional sites. Cell Cycle.

[177] Verlinden L., Vanden Bempt I., Eelen G., Drijkoningen M., Verlinden I., Marchal K., De Wolf-Peeters C., Christiaens M.R., Michiels L., Bouillon R. (2007). The E2F-regulated gene Chk1 is highly expressed in triple-negative estrogen receptor/progesterone receptor/HER-2 breast carcinomas. Cancer Res..

[178] Fischer M., Grossmann P., Padi M., DeCaprio J.A. (2016). Integration of TP53, DREAM, MMB-FOXM1 and RB-E2F target gene analyses identifies cell cycle gene regulatory networks. Nucleic Acids Res..

[179] Yang S.I., Lickteig R.L., Estes R., Rundell K., Walter G., Mumby M.C. (1991). Control of protein phosphatase 2A by simian virus 40 small-t antigen. Mol. Cell. Biol..

[180] Bollag B., Hofstetter C.A., Reviriego-Mendoza M.M., Frisque R.J. (2010). JC virus small T antigen binds phosphatase PP2A and Rb family proteins and is required for efficient viral DNA replication activity. PLoS ONE.

[181] Kwun H.J., Shuda M., Camacho C.J., Gamper A.M., Thant M., Chang Y., Moore P.S. (2015). Restricted protein phosphatase 2A targeting by Merkel cell polyomavirus small T antigen. J. Virol..

[182] Griffiths D.A., Abdul-Sada H., Knight L.M., Jackson B.R., Richards K., Prescott E.L., Peach A.H., Blair G.E., Macdonald A., Whitehouse A. (2013). Merkel cell polyomavirus small T antigen targets the NEMO adaptor protein to disrupt inflammatory signaling. J. Virol..

[183] Abdul-Sada H., Müller M., Mehta R., Toth R., Arthur J.S.C., Whitehouse A., Macdonald A. (2017). The PP4R1 sub-unit of protein phosphatase PP4 is essential for inhibition of NF-κB by merkel polyomavirus small tumour antigen. Oncotarget.

[184] Ramos F., Villoria M.T., Alonso-Rodríguez E., Clemente-Blanco A. (2019). Role of protein phosphatases PP1, PP2A, PP4 and Cdc14 in the DNA damage response. Cell Stress.

[185] Banerjee D., Langberg K., Abbas S., Odermatt E., Yerramothu P., Volaric M., Reidenbach M.A., Krentz K.J., Rubinstein C.D., Brautigan D.L. (2021). A non-canonical, interferon-independent signaling activity of cGAMP triggers DNA damage response signaling. Nat. Commun..

[186] Moens U., Passerini S., Falquet M., Sveinbjørnsson B., Pietropaolo V. (2023). Phosphorylation of Human Polyomavirus Large and Small T Antigens: An Ignored Research Field. Viruses.

[187] Schrama D., Hesbacher S., Angermeyer S., Schlosser A., Haferkamp S., Aue A., Adam C., Weber A., Schmidt M., Houben R. (2016). Serine 220 phosphorylation of the Merkel cell polyomavirus large T antigen crucially supports growth of Merkel cell carcinoma cells. Int. J. Cancer.

[188] Diaz J., Wang X., Tsang S.H., Jiao J., You J. (2014). Phosphorylation of large T antigen regulates merkel cell polyomavirus replication. Cancers.

